# Leptin Is Required for Glucose Homeostasis after Roux-en-Y Gastric Bypass in Mice

**DOI:** 10.1371/journal.pone.0139960

**Published:** 2015-10-07

**Authors:** Mohamad Mokadem, Juliet F. Zechner, Aki Uchida, Vincent Aguirre

**Affiliations:** 1 Fraternal Order of Eagles Diabetes Research Center, Roy J. and Lucille A. Carver College of Medicine, University of Iowa, Iowa City, Iowa, 52242, United States of America; 2 Division of Gastroenterology and Hepatology, Department of Internal Medicine, Roy J. and Lucille A. Carver College of Medicine, University of Iowa, Iowa City, Iowa, 52242, United States of America; 3 Division of Hypothalamic Research, Department of Internal Medicine, UT Southwestern Medical Center, Dallas, Texas, 75390, United States of America; 4 Division of Digestive and Liver Diseases, Department of Internal Medicine, UT Southwestern Medical Center, Dallas, Texas, 75390, United States of America; Institut d'Investigacions Biomèdiques August Pi i Sunyer, SPAIN

## Abstract

**Background & Aims:**

Leptin, the protein product of the *ob* gene, increases energy expenditure and reduces food intake, thereby promoting weight reduction. Leptin also regulates glucose homeostasis and hepatic insulin sensitivity via hypothalamic proopiomelanocortin neurons in mice. Roux-en-Y gastric bypass (RYGB) induces weight loss that is substantial and sustained despite reducing plasma leptin levels. In addition, patients who fail to undergo diabetes remission after RYGB are hypoletinemic compared to those who do and to lean controls. We have previously demonstrated that the beneficial effects of RYGB in mice require the melanocortin-4 receptor, a downstream effector of leptin action. Based on these observations, we hypothesized that leptin is required for sustained weight reduction and improved glucose homeostasis observed after RYGB.

**Methods:**

To investigate this hypothesis, we performed RYGB or sham operations on leptin-deficient *ob/ob* mice maintained on regular chow. To investigate whether leptin is involved in post-RYGB weight maintenance, we challenged post-surgical mice with high fat diet.

**Results:**

RYGB reduced total body weight, fat and lean mass and caused reduction in calorie intake in *ob/ob* mice. However, it failed to improve glucose tolerance, glucose-stimulated plasma insulin, insulin tolerance, and fasting plasma insulin. High fat diet eliminated the reduction in calorie intake observed after RYGB in *ob/ob* mice and promoted weight regain, although not to the same extent as in sham-operated mice. We conclude that leptin is required for the effects of RYGB on glucose homeostasis but not body weight or composition in mice. Our data also suggest that leptin may play a role in post-RYGB weight maintenance.

## Introduction

Recent randomized controlled trials proved that bariatric surgery is superior to conventional medical therapy for glycemic control in patients with diabetes mellitus [1, 2]. Roux-en-Y gastric bypass (RYGB) is the most commonly performed bariatric procedure in the U.S. and also one of the most efficacious for long-term sustained weight loss and resolution of multiple obesity-related co-morbidities [3, 4]. Understanding the mechanisms by which RYGB induces these beneficial effects will facilitate the development of therapies that are less invasive and can be applied more safely and broadly than surgery.

Leptin is secreted by adipocytes in proportion to adipose tissue mass and acts in the central nervous system to reduce energy intake and increase energy expenditure, thereby reducing body weight in states of positive energy balance [5–7]. During weight loss in humans and rodents, fat mass is reduced and plasma leptin levels fall. This triggers a complex set of metabolic and neuroendocrine adaptations including increased appetite and food intake as well as reduced energy expenditure, sympathetic nervous system tone, and circulating concentrations of bioactive thyroid hormone that favor weight regain and account for the high recidivism to obesity during dieting [8–14]. Leptin replacement to pre-weight-loss levels reverses this compensatory response [12, 15]. RYGB, on the other hand, induces extensive weight loss well beyond that needed (~10%) to trigger the above mentioned metabolic compensation [4, 9–12]. It also substantially reduces plasma leptin in patients [16], and in some studies beyond that observed in weight-matched and lean controls [17, 18]. Nonetheless, food intake and appetite are reduced, rather than increased, and weight loss is typically sustained long-term [4, 19]. Thus, patients after RYGB fail to manifest the expected compensatory response to reduced plasma leptin and instead exhibit behavioral and metabolic responses similar to weight-reduced patients (and rodents) receiving leptin replacement. These behavioral changes observed after RYGB seem more consistent with enhanced leptin signaling like improved leptin sensitivity for instance.

Leptin also exerts direct and indirect effects on glucose homeostasis [20, 21]. Despite equivalent weight loss post-RYGB, patients who fail to undergo remission of diabetes are hypoleptinemic when compared to those patients who do, as well as to lean controls [22].These lower levels of leptin in patients with sub-optimal diabetes response to RYGB suggest a neurohormonal role of leptin in mediating improvement in glucose metabolism In support of leptin’s role in RYGB, the full weight-reducing and gluco-regulatory effects of RYGB is melanocortin-4 receptor (MC4R)-dependent [23], a well-recognized mediator of many of leptin’s biological actions [24–30].

Therefore, we hypothesized that leptin is also involved in these effects of RYGB. To investigate this hypothesis, we performed RYGB or sham operations in leptin-deficient *ob/ob* mice, which are severely obese [31, 32], hyperinsulinemic, and insulin resistant [20, 21]. If our hypothesis is correct, RYGB will fail to reduce body weight and improve glucose homeostasis in *ob/ob* mice.

## Methods

### Animals

Care of all animals and procedures were approved by the UT Southwestern Medical Center Institutional Animal Care and Use Committee. Mice were housed in a temperature-controlled environment at 22°C-24°C using a 12 hour light/dark cycle (0600-1800 light) and provided water ad libitum. Lean C57BL/6 mice (stock#: 000664), DIO C57BL/6 mice (stock#: 380050) and *ob/ob* mice (stock#: 000632) were purchased from the Jackson Laboratory (Bar Harbor, ME). *ob/ob* mice were provided regular chow (RC) comprised of 22%/66%/12% calories from protein/carbohydrate/fat (2016, Teklad Diets, Madison, WI) and DIO mice provided high fat diet (HFD) comprised of 20%/20%/60% calories from protein/carbohydrate/fat (D12492, Research Diets, Inc., New Brunswick, NJ) ad libitum, unless otherwise specified.

### Surgery

RYGB was performed as previously described [23]. In brief, RYGB involved Roux-en-Y reconstruction of the gastrointestinal tract after transection of the intestine immediately distal to the ligament of Trietz via primary anastomosis connecting the distal transected jejunal limb to the proximal anterior gastric wall and the proximal transected jejunal limb to the afferent Roux limb. The proximal gut was excluded from alimentary flow using a hemostasis clip (Ethicon Endosurgery, Cincinnati, OH) placed immediately distal to the gastro-jejunostomy. The sham procedure involved gastrotomy, enterotomy, and repair. Anesthesia was provided using a scavenged circuit of isoflurane and anesthesia time was standardized between groups. All mice were maintained on a standardized post-operative protocol during which liquid diet (Vital HN, Abbott Labs) was provided from post- operative day 2-7. Solid diet was re-introduced starting on post-operative day 6.

### Study design

At 10 weeks of age, *ob/ob* mice were randomized to RYGB or sham operations. As we have previously observed [23], both surgical procedures induce substantial post-operative weight loss (typically ~30%) due to intra-operative stress and peri-operative calorie restriction as observed at the end of post-operative week 1. To avoid confounding of RYGB-induced changes in energy balance and body weight by this peri-operative weight loss between animals and groups, all mice were weight-matched to the RYGB group via calorie restriction during week 2. At the end of week 2, RYGB and sham mice were provided RC ad libitum. Two additional cohorts of *ob/ob* mice were randomized to sham-procedures; one cohort were pair-fed to RYGB mice from the end of week 2 through the end of the study (PF-sham) and the other was weight-matched via daily calorie restriction (WM-sham). PF-sham animals were provided a pre-determined amount of diet equivalent to the average consumed by RYGB animals (of the same age and post-operative days) for the previous 24-hour period. WM-sham animals were provided a calorie-restricted meal to maintain body weight at an equivalent % body weight loss to RYGB animals (of the same age and post-operative days). Amount of calories provided to WM-sham animals was determined on a daily basis by weight change and calories provided in the previous 24-hour period. All calorie restricted animals were provided their restricted calories in a single meal immediately prior to the start of the dark cycle. Body weight was monitored weekly and body composition measured using the Bruker Minispec mq10 NMR (Bruker Optics, Billerica, MA) during weeks 2 and 5. To avoid confounding metabolic effects of post- operative convalescence, glucose homeostasis was evaluated during weeks 6 through 9. Food intake was measured serially throughout weeks 2 through 5. DIO C57BL/6 mice were randomized to RYGB and sham operations upon reaching 45g, which occurred at approximately 16-19 weeks of age. DIO mice received the same post-operative feeding protocol for the first week as mentioned above for ob/ob mice; however, due to their previously reported faster recovery of food intake behavior (day 8–10) these mice were put on ad-libitum HFD feeding as of day 8.

### Energy Balance

Total 24-hr energy expenditure was estimated using an energy balance technique in which change in energy stores are subtracted from energy intake over time. This methodology has been validated versus indirect calorimetry for the accurate measure of long-term changes in energy expenditure [33]. Body weight and food intake were measured serially from the end of post-operative week 2 through the end of week 5. Metabolizable energy intake, defined as grams of solid diet ingested per day multiplied by 12.57 kJ/g, was calculated from the food intake measurements. Feeding efficiency was calculated by dividing weight change (in mg) by food consumed (in kJ). In this methodology, mean total 24-hr energy expenditure (kJ/d) represents the consumed energy remaining for physical activity and basal metabolism after accounting for changes in body mass [33–36]. The energetic cost of fat mass and lean mass deposition was estimated using values of 55.3 kJ/g and 9.2 kJ/g, respectively and the energy contents of fat and lean mass were estimated as 37.7 kJ/g and 4.2 kJ/g, respectively [36, 37]. When animals lost weight, there was no energetic cost of deposition and the body energy lost was subtracted from the energy equation [33–35].

### HFD Challenge

Ad libitum-fed sham and RYGB-treated *ob/ob* mice were challenged with HFD for 8 days during post-operative week 7-8. Food intake and body weight were measured on a daily basis. Metabolizable energy intake, defined as grams of solid diet ingested per day multiplied by 22.0 kJ/g, was calculated from the food intake measurements.

### Fasting blood glucose

Following an overnight fast (1800-1000, during post-operative weeks 6–9), blood glucose was measured from tail vein blood using a hand-held glucometer (Contour, Bayer Healthcare, Tarrytown, NY).

### Glucose tolerance test

Following an overnight fast (1800-1000, during post-operative weeks 6–9), mice were administered a 1g/kg BW bolus of D-glucose (G82070, Sigma, St. Louis, MO) by oral gavage. Blood glucose was measured from tail vein blood using a hand-held glucometer (Contour) immediately before and 15, 30, 60, and 120 minutes after glucose administration.

### Insulin tolerance test

Following a 4-hour fast (0900-1300, during post-operative weeks 6–9), mice were administered a 0.75U/kg BW dosage of insulin (HumulinR, Eli Lilly and Co., Indianapolis, IN) by intraperitoneal injection. Blood glucose was measured from tail vein blood using a hand-held glucometer (Contour) immediately before and 15, 30, and 60 minutes after injection. Insulin tolerance data is presented as curves normalized to baseline glucose levels.

### Fasting and glucose-stimulated plasma hormones

Plasma samples were collected from the tail vein of overnight fasted mice as well as 15 and 30 minutes after glucose administration by oral gavage (1g/kg BW, during post-operative weeks 6–9). Insulin levels were measured using the Ultra-Sensitive Mouse Insulin ELISA Kit from Crystal Chem Inc. (Downers Grove, IL). Glucagon levels were measured with assistance from the laboratory of Dr. Roger Unger, UT Southwestern Medical Center.

### Tissue Chemistries

Triglyceride levels were measured from frozen liver using a colorimetric method by the UT Southwestern Medical Center Mouse Metabolic Phenotyping Core.

### Statistical Analysis

All data are presented as mean ± standard error of the mean. Only *P*-values less than .05 were considered statistically significant. Experiments comparing two means were analyzed using Student’s t-test, with Welch’s correction as appropriate; experiments comparing three or more means were analyzed using one-way analysis of variance (followed by Tukey Post-Hoc). RYGB-treated *ob/ob* mice were compared to age-matched, lean C57BL/6 or DIO C57BL/6 mice using Student’s t-test (with Welch’s correction, when appropriate). Curves were analyzed using both repeated measures two-way analysis of variance and area-under-curve analysis (trapezoidal rule) using Student’s t-test or one-way analysis of variance (followed by Tukey Post-Hoc), where appropriate.

## Results

### RYGB reduces body weight in leptin-deficient ob/ob mice

**Fig 1:**
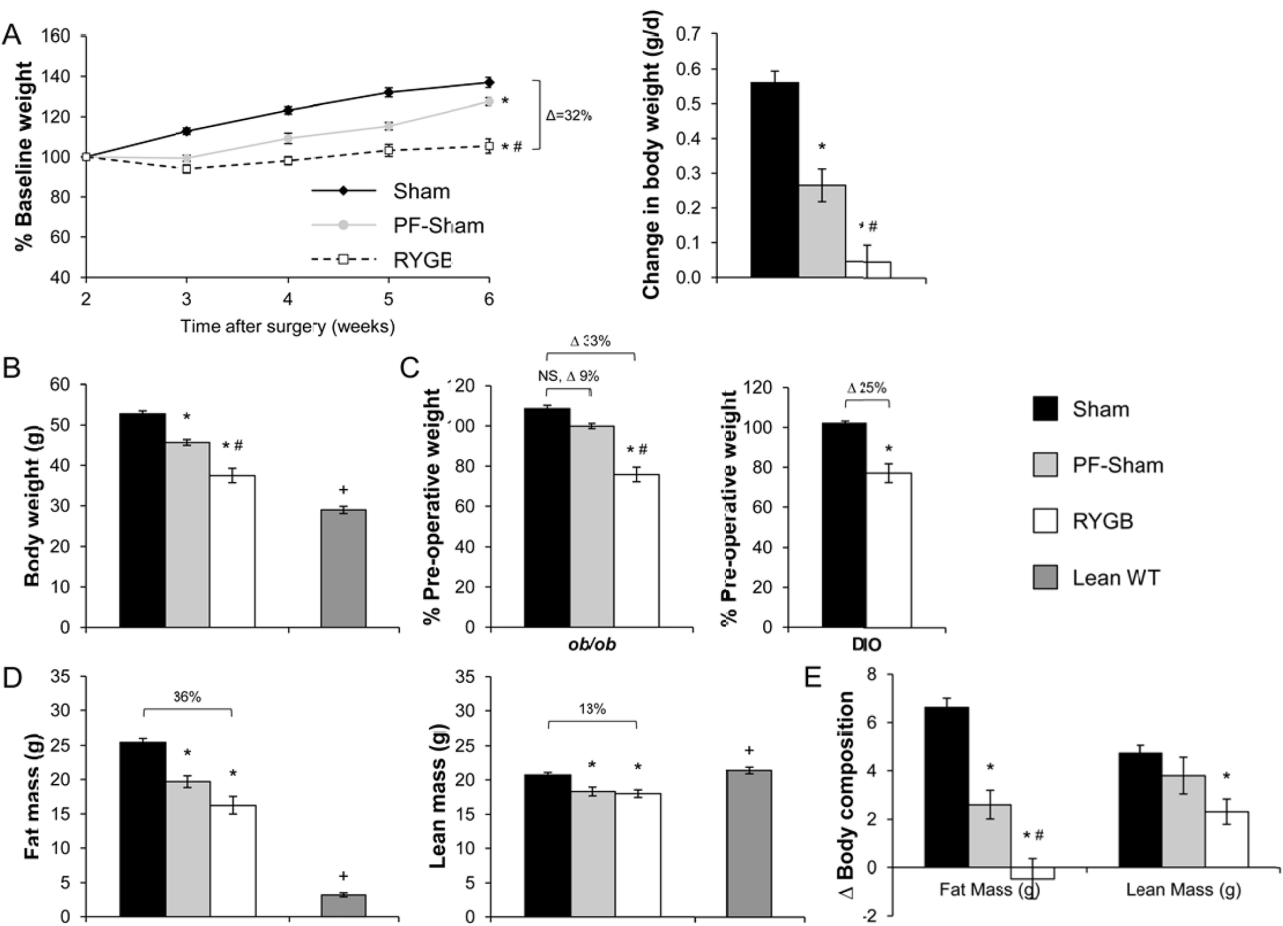
RYGB induces weight loss in *ob/ob* mice. (A, Left) Body weight curves expressed as a percentage of body weight at post-operative week 2 (upon resumption of ad libitum diet after post-operative recovery) for sham, PF-sham, and RYGB-treated *ob/ob* mice from post-operative week 2 through 6. (A, Right) Average daily change in body weight during this same period. (B) Total body weight as measured during week 6. (C) Total body weight during week 6, expressed as a percentage of pre-operative weight in *ob/ob* mice. DIO mice are presented as a control for the effects of surgery on body weight. (D) Fat and lean mass in sham, PF-sham, and RYGB-treated *ob/ob* mice as measured during week 5. (E) Change in body composition (fat and lean mass) in sham, PF-sham, and RYGB-treated *ob/ob* mice from week 2 to week 5. Non-operated, age-matched C57BL/6 mice (Lean WT) maintained on regular chow are presented in panels (B) and (D) as controls for the expected body weight, fat mass, and lean mass of age-matched lean mice. (*ob/ob*: n = 21–25, sham; n = 7, PF-sham; n = 17, RYGB |Lean WT: n = 5 | DIO WT: n = 7, sham; n = 8, RYGB). Results are presented as mean ± SEM. * *P <* .05 vs sham, # *P <* .05 vs PF-sham, + *P <* .05 lean vs RYGB. Curves of Panel (A) were assessed using 2-Way ANOVA with repeated measures. All other comparisons were assessed using 1-Way ANOVA or Student’s t-test, where appropriate.

Both surgical groups lost weight due to the effects of intra-operative stress and peri-operative calorie restriction. After post-operative recovery and upon resumption of ad libitum (RC) at the end of post-operative week 2, sham-operated animals gained 0.56±0.03 g/day, reaching a total body weight of 52.7±0.8g by week 6 (Fig 1A and 1B). By contrast, RYGB mice regained weight at a greatly reduced rate of 0.05±0.05 g/day and reaching only 37.5±1.8g by week 6, a reduction of 29% compared to shams (Fig 1A and 1B). Expressed as a percentage of pre-operative weight, sham mice weighed 108.6±1.7% while RYGB mice weighed 75.8±3.6% for a difference of 33% (Fig 1C, left). RYGB reduced fat mass by 36% and lean mass by 13% (Fig 1D). During week 6, the total body weight of sham-treated *ob/ob* mice was equivalent to non-operated, age-matched *ob/ob* mice (52.7±0.8 g, sham-treated *ob/ob* mice versus 55.3±0.6 g, non-operated *ob/ob* mice, p>.05) demonstrating complete recovery from their peri-operative weight loss. During this week, RYGB-treated *ob/ob* mice also weighed 29% more (total body weight) than a non-operated, age-matched lean C57BL/6 mouse (37.5±1.8 g, RYGB versus 29±0.9 g, lean C57BL/6, *P* < .05), a difference due predominately to fat mass (Fig 1B and 1D). RYGB reduced the total body weight of diet-induced obese (DIO) C57BL/6 mice to a similar extent as observed in *ob/ob* mice (Fig 1C, right). However, in contrast to *ob/ob* mice, the total body weight of RYGB-treated DIO C57BL/6 mice (Fig 1C, right) was equivalent to that observed in non-operated, age-matched lean C57BL/6 mice (35.6±2.1g, RYGB versus 33.0±1.3g, lean C57BL/6, *P* >.05).

### RYGB reduces energy intake and feeding efficiency in ob/ob mice

**Fig 2:**
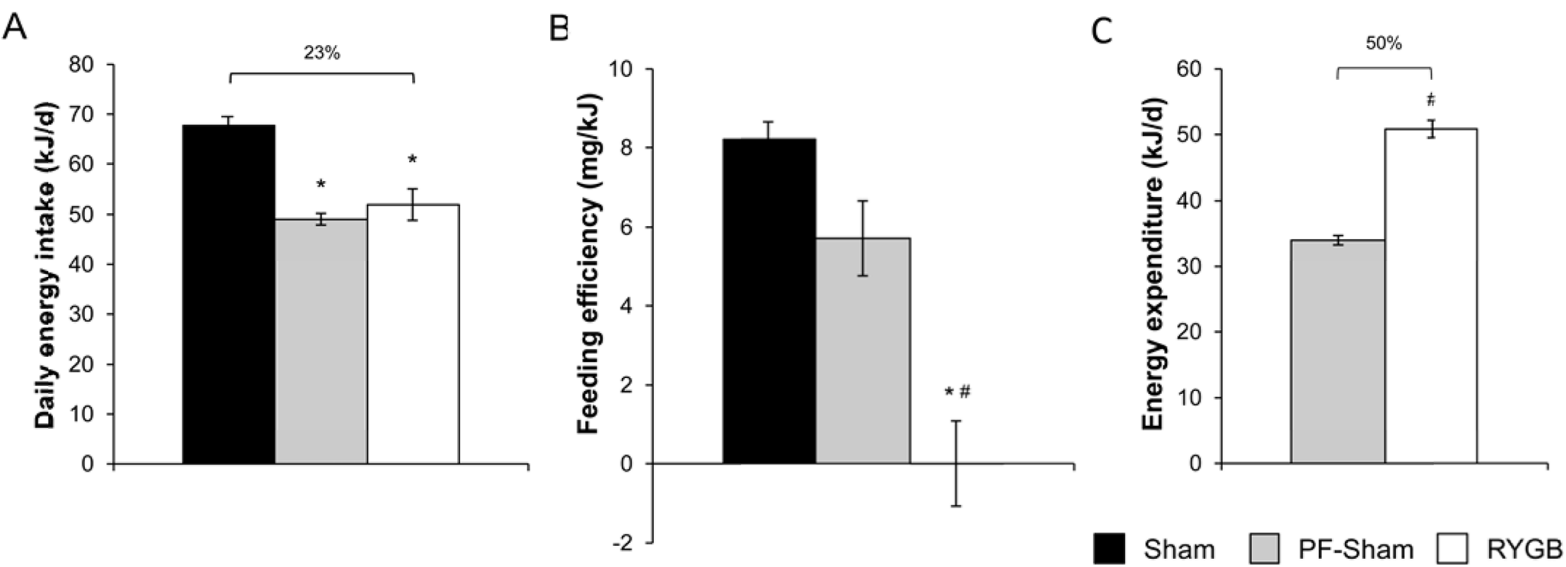
RYGB reduces energy intake feeding efficiency in *ob/ob* mice. (A) Daily energy intake (in kJ) in sham, PF-sham and RYGB-treated *ob/ob* mice. (B) Feeding efficiency (weight change per kJ of diet consumed) in sham, PF-sham and RYGB-treated *ob/ob* mice. (C) Total 24-hr energy expenditure (in kJ/day) in PF-sham and RYGB. (n = 24–25, sham; n = 7, PF-sham; n = 17, RYGB). Results are presented as mean ± SEM. * *P <* .05 vs sham, # *P <* .05 vs PF-sham were assessed using 1-Way ANOVA or Student’s t-test, where appropriate.

RYGB reduced daily energy intake of RC by 23% compared to sham surgery (Fig 2A). To investigate the contribution of this reduced intake to RYGB-induced weight loss, a separate cohort of sham-operated mice were pair-fed to the daily intake of RYGB mice (PF-sham) from week 2 through the end of the study. Compared to the 33% difference in normalized body weight observed between RYGB and sham mice during week 6, the difference between PF-sham and sham was only 9% and not statistically significant (99.9±1.3%, PF-sham versus 108.6±1.7%, sham, *P* >.05; Fig 1C). As total body weight, PF-shams weighed 13% less than shams (45.7±0.7 g, PF-sham versus 52.7±0.8 g, sham, *P* < .05; Fig 1B) while RYGB mice weighed 29% less. PF-shams gained more weight as fat mass than RYGB mice while gain in lean mass was equivalent (Fig 1E). The greater weight reduction of RYGB mice compared to PF-shams was due to the effect of RYGB to reduce feeding efficiency (weight gain per kJ consumed) compared to both sham and PF-sham mice (Fig 2B). These observations therefore suggest that RYGB augments basal metabolism. Consistent with this hypothesis, estimation of energy expenditure using an energy balance technique demonstrates that 24-hr total energy expenditure is increased in RYGB mice compared to PF-shams (Fig 2C). These data demonstrate that leptin is dispensable for RYGB-induced weight reduction due to the preserved effect of RYGB to reduce food intake and feeding efficiency in *ob/ob* mice.

### HFD promotes positive energy balance and weight gain in RYGB-treated ob/ob mice

**Fig 3:**
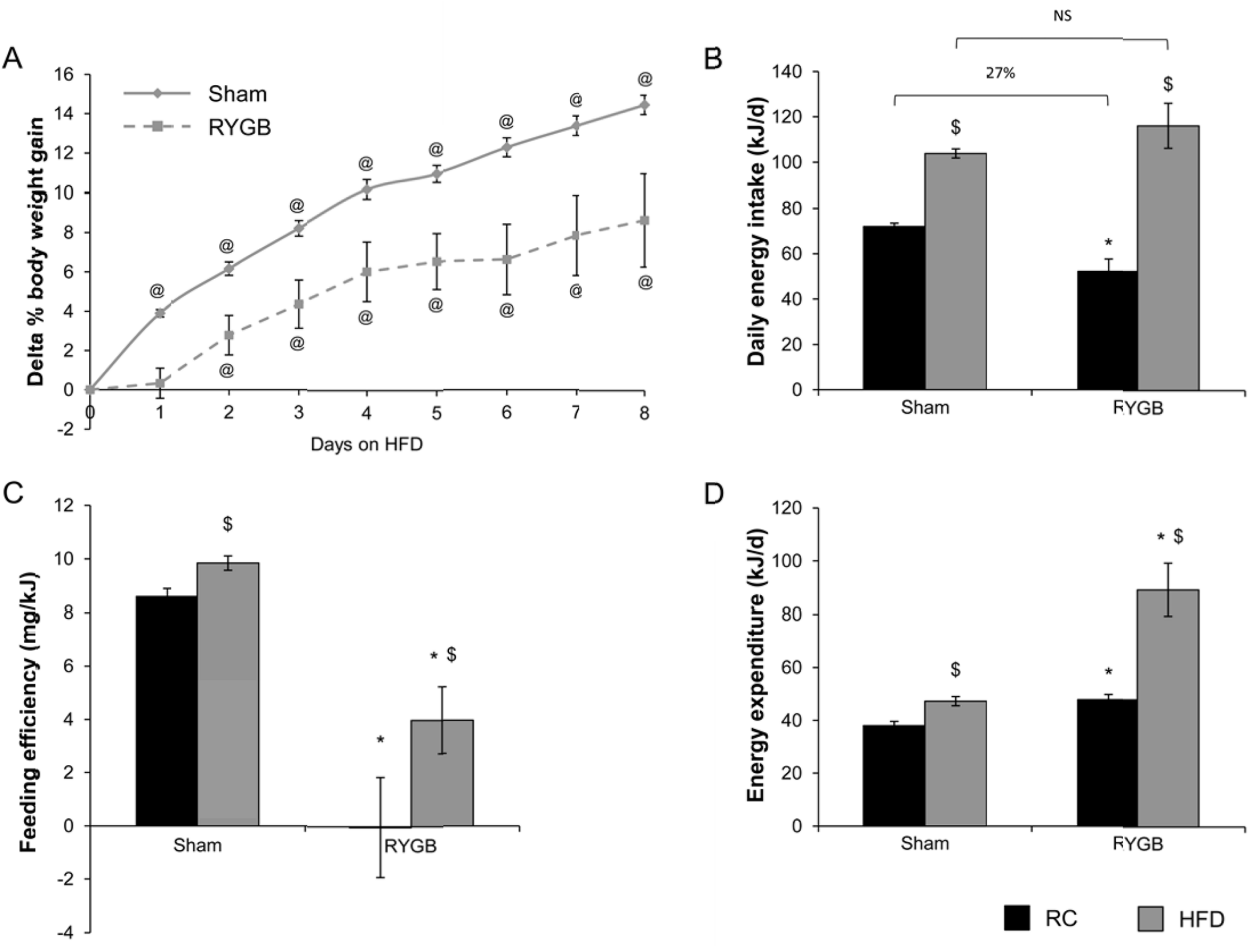
High fat diet promotes positive energy balance and weight gain in RYGB-treated *ob/ob* mice. (A) Daily weight gain during challenge of sham and RYGB-treated *ob/ob* mice with high fat diet (HFD), expressed as a percentage of pre-challenge body weight. (B) Average daily energy intake on regular chow (RC) and during challenge with HFD in sham and RYGB mice. (C) Feeding efficiency and (D) energy expenditure in sham and RYGB mice on RC and HFD. (n = 15, sham; n = 8, RYGB). Results are presented as mean ± SEM. ^@^ *P <* .05 vs day 0 within surgical group of panel (A), and $ *P <* .05 vs RC within surgical group or *P < .05 vs sham within diet group for panels (A, B, C) were assessed using Student’s t-test.

To determine whether RYGB-treated *ob/ob* mice on RC defend their neutral energy balance (i.e., no or minimal weight change per kJ of diet consumed) and stable reduced body weight (Figs 1A and 2B), we challenged RYGB and sham *ob/ob* mice on RC with HFD. Upon challenge with HFD, sham-treated *ob/ob* mice significantly increased their total body weight in comparison to their pre-challenge weight by the end of the first day of the study (Fig 3A). This effect was delayed in RYGB-treated *ob/ob* mice, reaching significance at the end of the second day (Fig 3A). This delay resulted in a difference between the post-challenge weight gains of sham and RYGB mice that was maintained for the duration of the experiment (Fig 3A). While daily intake on HFD was increased in both surgical groups, the increase was greatest in RYGB mice (Fig 3B). In contrast to the anorectic effect of RYGB observed on RC, daily intake of HFD was equivalent between RYGB and sham mice (Fig 3B). Feeding efficiency was also increased in both surgical groups (Fig 3C). However, overall efficiency was still lower in RYGB mice compared to shams accounting for the difference in weight gain (Fig 3C). This reduced feeding efficiency was due to a greater effect of RYGB-treated *ob/ob* mice to increase total energy expenditure in response to challenge with HFD (Fig 3D). Thus, HFD successfully promotes promote positive energy balance and weight gain in RYGB-treated *ob/ob* mice, albeit to a lesser degree than in sham *ob/ob* mice.

### RYGB fails to improve glucose homeostasis in ob/ob mice

**Fig 4:**
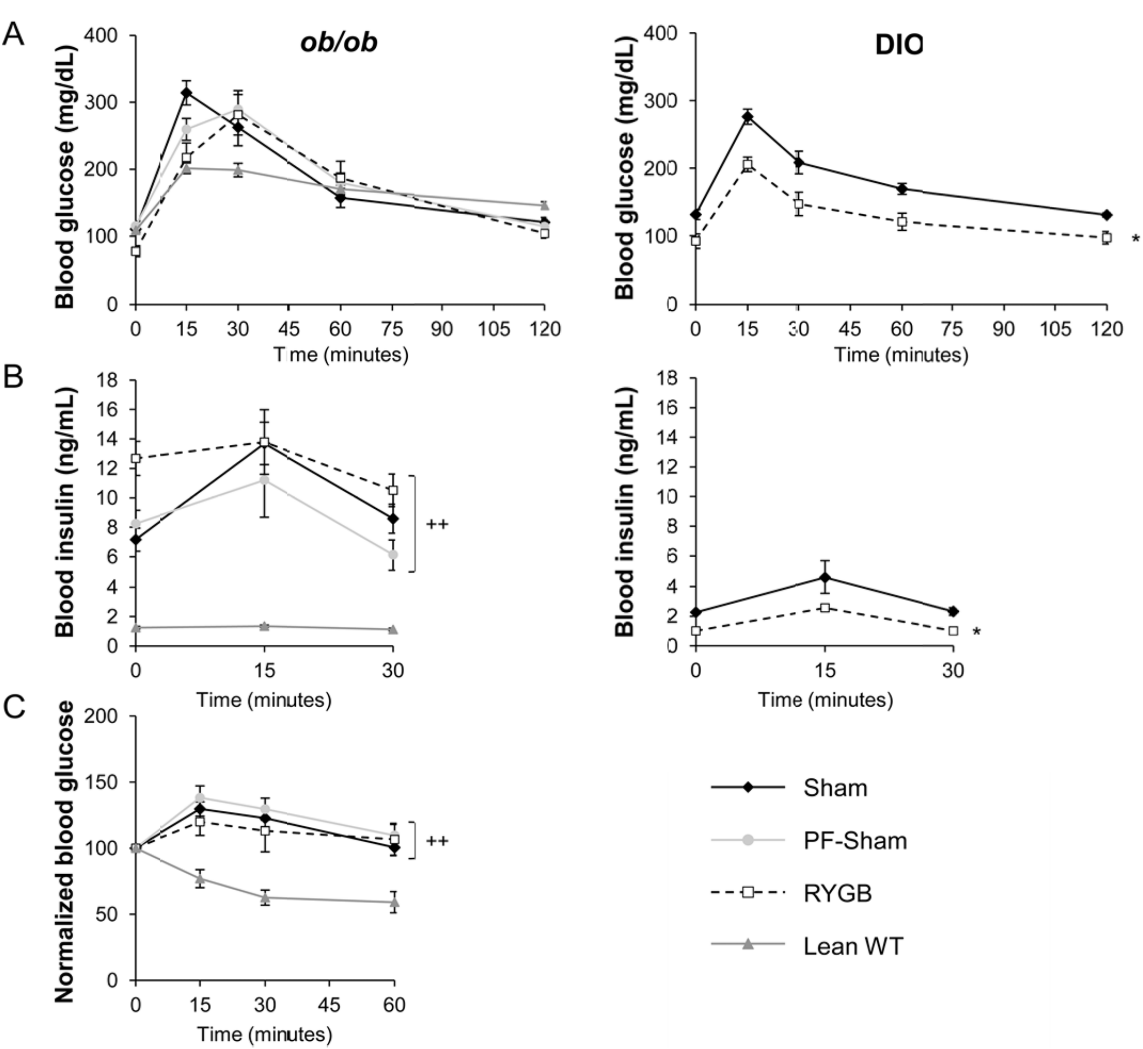
RYGB fails to improve glucose and insulin tolerance as well as glucose-stimulated plasma insulin in *ob/ob* mice. (A, left) Glucose tolerance (B, Left) Glucose-stimulated plasma insulin and (C) insulin tolerance in sham, PF-sham, and RYGB-treated *ob/ob* mice. Non-operated, age-matched, lean C57BL/6 mice (Lean WT) are presented as control for insulin sensitivity. Data from sham and RYGB DIO mice are shown in panels (A, Right) and (B, Right) as controls for the effects of surgery on glucose homeostasis. (*ob/ob*: n = 21, sham; n = 7, PF-sham, n = 13–15, RYGB | Lean WT: n = 6–12 | DIO: n = 5–7 sham, n = 6–8, RYGB). Results are presented as mean ± SEM. Curves were analyzed using area-under-the-curve analysis. ^++^ p < .05 vs lean assessed using 1-Way ANOVA for *ob/ob* mice and * p < .05 vs sham assessed using Student’s t-test for DIO mice.

RYGB failed to improve glucose tolerance or reduce glucose-stimulated plasma insulin levels in *ob/ob* mice (Fig 4A and 4B, left panel). RYGB also failed to improve insulin tolerance (Fig 4C). These parameters were improved in DIO C57BL/6 mice after RYGB (Fig 4A and 4B, right panel). Age-matched, lean C57BL/6 mice on regular chow were studied in parallel as controls (Fig 4A–4C, left). These data suggest that leptin is required for the effect of RYGB to improve peripheral insulin sensitivity.

**Fig 5:**
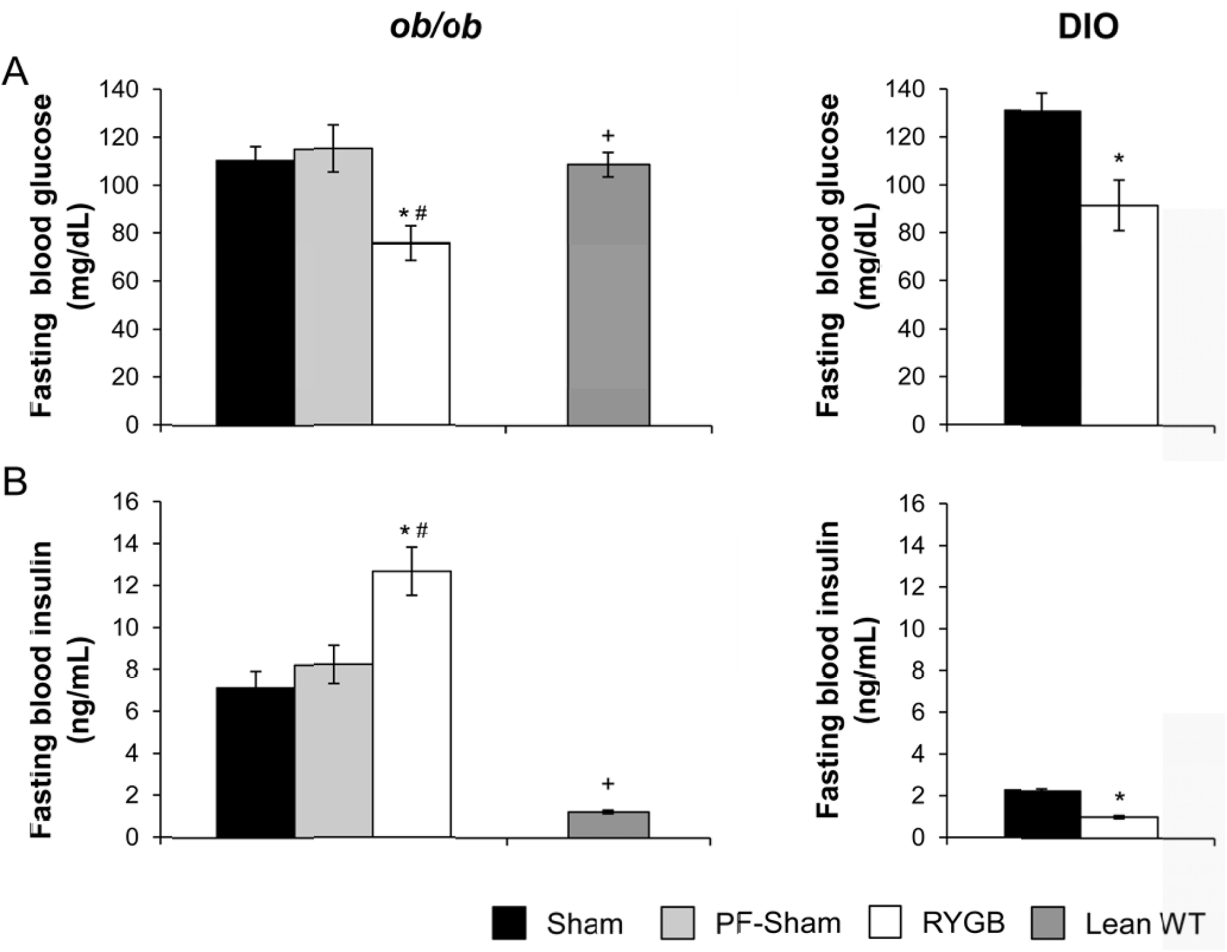
RYGB reduces fasting glucose in *ob/ob* mice. (A, left) Fasting blood glucose and (B, Left) fasting blood insulin in sham, PF-sham, and RYGB-treated *ob/ob* mice. Non-operated, age-matched, lean C57BL/6 mice (Lean WT) are presented as control for insulin sensitivity. Data from sham and RYGB-treated DIO mice are shown in panels (A, Right) and (B, Right) as controls for comparison. (*ob/ob*: n = 21, sham; n = 7, PF-sham, n = 13–15, RYGB | Lean WT: n = 6–12 | DIO: n = 5–7 sham, n = 6–8, RYGB). Results are presented as mean ± SEM. * p < .05 vs sham, ^#^ p < .05 vs PF-sham, ^+^ p < .05 lean vs RYGB were assessed using 1-Way ANOVA or Student’s t-test, where appropriate.

In contrast, RYGB reduced fasting blood glucose compared to shams and PF-shams in *ob/ob* mice (Fig 5A, left). Fasting plasma insulin was increased after RYGB (Fig 5B, left) while plasma glucagon was unchanged (data not shown). In a separate experiment, a cohort of sham-treated *ob/ob* mice was weight-matched to RYGB-treated *ob/ob* mice (WM-sham) by calorie restriction to determine if the reduced fasting glucose observed in RYGB-treated *ob/ob* mice was a secondary consequence of weight loss. Fasting blood glucose was reduced in RYGB-treated mice compared to WM-shams (68±13 mg/dL, RYGB versus 130±5 mg/dL, WM-sham; P < .05) despite equivalent fasting plasma insulin (10.22 ± 0.28 ng/mL, RYGB versus 14.04 ± 1.71 ng/ml, WM- sham; *P* >.05). Fasting plasma glucagon was increased in RYGB mice compared to WM-shams (88.3±13.6 pg/ml, RYGB versus 43.7±3.6 pg/ml, WM-sham; *P* < .05). As expected, RYGB reduced both fasting blood glucose and plasma insulin in DIO C57BL/6 mice (Fig 5A and 5B, right panels) while fasting plasma glucagon was unchanged (data not shown). These data demonstrate that the effect of RYGB to reduce fasting glucose is independent of changes in body weight and food intake as well as plasma leptin, insulin, and glucagon.

### RYGB reduces hepatic triglyceride content in ob/ob mice

**Fig 6:**
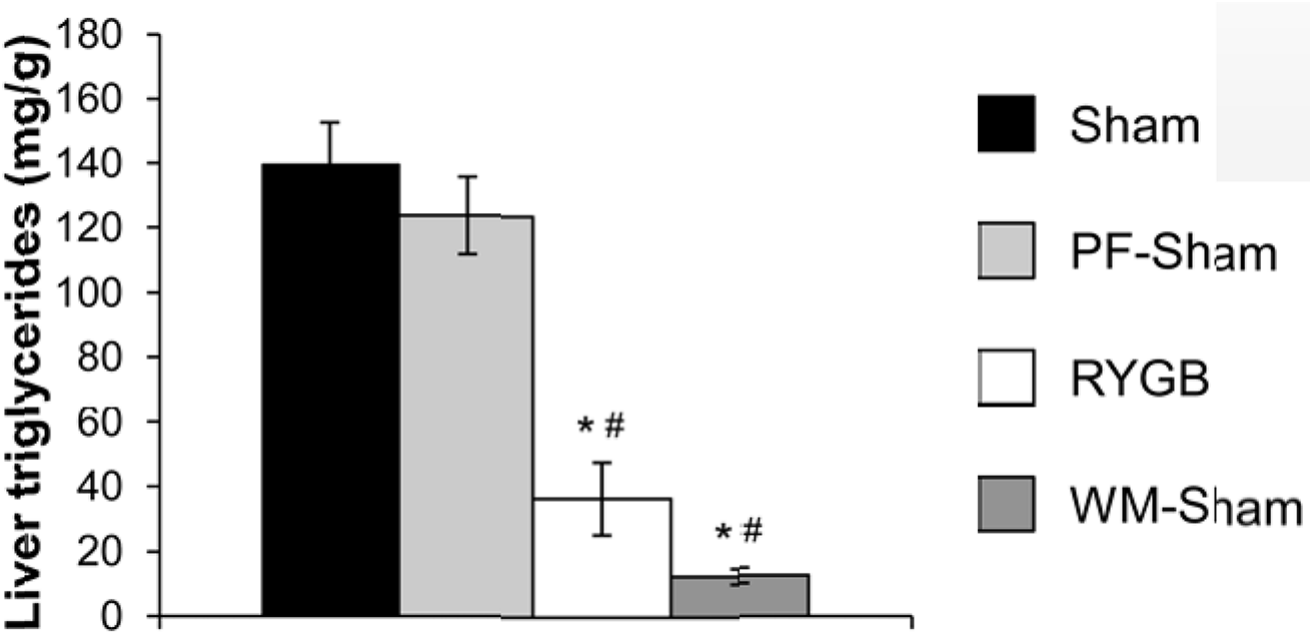
Leptin is not required for effects of RYGB on fasting hepatic triglyceride. Total triglyceride content in liver of sham, PF-sham, RYGB, and WM-sham *ob/ob* mice. (n = 7, sham; n = 7, PF-sham, n = 3, RYGB, n = 7, WM-sham). Results are presented as mean ± SEM. * p < .05 vs sham, ^#^ p < .05 vs PF-sham were assessed using 1-Way ANOVA.

To investigate if leptin is required for the effect of RYGB to reduce hepatic triglyceride content, we measured total triglyceride content in livers of fasted sham, PF-sham, RYGB, and WM-sham *ob/ob* mice. RYGB reduced total hepatic triglyceride content compared to sham and PF-sham mice (Fig 6). Hepatic triglyceride content was similarly reduced in WM-sham mice (Fig 6). Thus, the effect of RYGB to reduce hepatic triglyceride does not require leptin.

## Discussion

In this manuscript, we demonstrate that the effects of RYGB to reduce body weight and fat mass are preserved in leptin-deficient *ob/ob* mice maintained on RC. In these mice, RYGB reduces energy intake and increases energy expenditure to cause weight loss, demonstrating that these biological effects of RYGB are leptin-independent. We have previously demonstrated that despite being maintained on HFD after surgery, RYGB operated DIO C57BL/6 mice had similar body weights to age-matched, non-operated lean C57BL/6 control mice [38]. While the percent reduction of body weight seen after RYGB treatment of *ob/ob* and DIO C57BL/6 mice is comparable (~30% versus shams), RYGB fails to reduce the body weight of *ob/ob* mice to the same level of age-matched, C57BL/6 lean controls. Moreover, *Hao et al*. showed that *ob/ob* mice (at different age groups) regained their pre-operative weights 8 weeks after gastric bypass while DIO C57BL/6 mice did not. These RYGB-treated *ob/ob* mice; however, were still significantly leaner than their respective shams [39]. This suggests that leptin is only required for a portion of the weight-reducing effects of RYGB. Unfortunately, it is not possible to perform a direct comparison of RYGB effects in *ob/ob* and wild-type mice on RC because, in our experience, WT mice do not become sufficiently obese to withstand the peri-operative stress of RYGB.

The weight loss observed after RYGB in patients and rodents is not only substantial, but sustained.

Thus, while deemed dispensable for induction of weight loss, the long-term weight maintenance observed after RYGB in patients and rodents suggest that leptin may be involved in defense of this new, reduced post-surgical body weight. Consistent with our hypothesis, RYGB-treated *ob/ob* mice gained weight when challenged with HFD, albeit not to the same extent as shams. Leptin-deficient ob/ob mice have innate hyperphagia on RC which was significantly improved after RYGB but may not be completely resolved. It’s possible that RYGB-treated *ob/ob* mice are still somewhat hyperphagic on RC, especially since they do not normalize their body weight and fat mass to level of age-matched lean C57BL/6 controls like RYGB-treated DIOs [38]. Unfortunately, as mentioned above, we were unable to have the appropriate controls to address this question as wild-type C57BL/6 mice do not gain sufficient weight on RC to tolerate RYGB. It was previously shown that *ob/ob* mice significantly increase their body weight and their daily calorie consumption when placed on HFD [40]. We observed a similar finding when sham-treated *ob/ob* mice increased their daily energy consumption when placed on HFD and not given the chance to choose between diets. Interestingly, RYGB-treated *ob/ob* mice also increased their energy consumption on HFD to the same level of their sham counterparts. This similar food intake behavior between RYGB and sham-treated *ob/ob* mice while on HFD was previoulsy observed in DIO mice fed the same HFD [38].Therefore, while RYGB can reduce food intake in leptin-deficient mice on RC, it fails to maintain this reduction on HFD.

We previously showed, via bomb calorimetry, that the difference in caloric absorption between RYGB and sham-operated mice was ~7–11%. This small difference in absorptive efficiency of the gut seems to minimally contribute to the large weight reduction effect of gastric bypass (~ 30% difference in weight between groups) [23, 38]. Based on this observation we used a validated method to calculate energy expenditure [33] assuming that the contribution of calorie absorption to energy balance after RYGB is somehow negligible. As physiologically expected, sham-treated *ob/ob* mice increase, though not tremendously, their energy expenditure to resist weight gain imposed by the positive energy balance of the HFD. After RYGB, however, *ob/ob* mice experience a more pronounced increase in energy expenditure on HFD; therefore, better defending their weights compared to their sham counterparts that are consuming similar amount of calories (Fig 3A). Nonetheless, this RYGB-induced increase in energy expenditure of *ob/ob* mice while on HFD seems insufficient to maintain their neutral weight balance observed while on RC (Fig 3C and 3D). The effect of RYGB-treated mice to defend a reduced body weight on RC requires MC4R, a downstream mediator of many of leptin’s biological actions. This is demonstrated by the fact that both RYGB-treated and sham-treated MC4R- deficient mice gain substantial and equivalent weight when challenged with HFD [23]. These observations suggest that leptin is also involved in the effect of RYGB to sustain reduced weight and intake, at least on RC. As these effects are lost or mitigated on HFD, this suggests that augmented leptin-melanocortin signaling may play an adjunctive therapeutic role for patients with weight regain after RYGB, which is typically associated with increased intake and poor dietary adherence [41]. This hypothesis needs to be formally tested in the pre- clinical and clinical setting.

Despite the preserved effects of RYGB to reduce body weight and improve body composition in *ob/ob* mice, it failed to improve glucose tolerance, insulin tolerance, or reduce fasting and glucose- stimulated plasma insulin. *Hao* et al. also reported a lack of improvement in fasting insulin level and oral glucose tolerance in *ob/ob* mice after gastric bypass and these parameters improved with leptin administration [39]. Direct effects of leptin on glucose homeostasis that are independent of changes in food intake, body weight, body composition, plasma insulin, and plasma glucagon have been well- characterized [20, 21]. Notably, central leptin administration improves insulin-deficient diabetes in rodents independent of leptin-induced reduction in body weight and food intake [42, 43], improves glucose homeostasis in obese mice independent of leptin-induced changes in body weight [44], and has acute effects on hepatic and peripheral glucose flux [45–48]. In addition, genetic and acquired lipoatrophic rodents and patients are leptin-deficient, hyperglycemic, and glucose intolerant despite being hyperinsulinemic, further demonstrating a dissociation of leptin, insulin, body weight, and glucose control [49–51]. The likely cause of at least a subset of these gluco-regulatory effects of leptin is the vagal control of hepatic insulin sensitivity [52], a feature that we have described for weight-independent improved gluco-regulation after RYGB occurring through MC4Rs in autonomic brainstem neurons [23]. Thus, the fact that RYGB can induce substantial weight reduction but not improve glucose control in leptin-deficient *ob/ob* mice is not entirely unexpected. Consistent with our findings in mice, a relative leptin deficiency has been associated with lack of remission of type 2 diabetes after RYGB in patients exhibiting the expected post-surgical weight loss [22].

The fact that RYGB-induced reduction in fasting blood glucose is preserved in *ob/ob* mice is surprisingly interesting. This effect is independent of reduced intake, as it did not occur in PF-sham mice. It is also independent of changes in body weight, gluco-regulation by glucagon, and hepatic triglyceride content, as it did not occur in WM-sham mice. One possible explanation of this improved fasting blood glucose in RYGB-treated *ob/ob* mice could be the observed increase in fasting blood insulin (Fig 5A and 5B). We previously showed that both fasting and glucose-stimulated GLP–1 levels increase significantly after gastric bypass [38]. This RYGB-induced increase in GLP–1 secretion (and possibly other incretins) might be one of the reasons behind the increase in fasting blood insulin. This exaggerated increase in fasting insulin level in RYGB-treated *ob/ob* animals compared to RYGB-treated DIOs is likely due to their persistent peripheral insulin resistance. We did not measure serum GLP–1 levels in our *ob/ob* mice after RYGB but we did not have reasons to believe that GLP–1 secretion is compromised in *ob/ob* mice after surgery although this possibility is not ruled out.

Although we did not conduct studies to specifically address these points, it is of note that the timing of the peak glucose levels observed in RYGB-treated *ob/ob* mice during oral tolerance test was delayed (occurring at 30 minutes) compared to usual glucose peak we see in wild-type DIO and lean mice (occurring at 15 minutes). Possible explanations for this delayed glucose peak could be (i) a delay in incretin-induced insulin surge or (ii) a decrease in peripheral glucose uptake rate after RYGB under leptin-deficient conditions or (iii) changes in gut hormones secretion pattern (like GLP–1, CCK and P-YY) affecting gastric emptying, intestinal motility and, therefore, glucose absorption. These findings suggest a segregation of the effect of gastric bypass between fasting and non-fasting glucose metabolism possibly via a direct neuronal effect of RYGB to reduce hepatic glucose production. Consistent with this hypothesis is our finding that MC4Rs in autonomic brainstem neurons mediate direct effects of RYGB to improve hepatic glucose output, as measured using pyruvate tolerance testing, in a manner independent of RYGB-induced changes in food intake, body weight, and body composition [23]. Furthermore, an intriguing alternative hypothesis for improved glycemia after RYGB that is independent of changes in body weight and plasma insulin is the re-programming of intestinal glucose metabolism and glycemic control recently proposed to occur after RYGB in rats [53]. It is currently unknown if this mechanism contributes to improved glucose in mice and humans. Our data are complementary to previous studies reporting preserved effects of RYGB on body weight and glucose homeostasis in Zucker rats [54–57]. Zucker rats carry the autosomal recessive *fa* mutation of the leptin receptor, a missense mutation of the extracellular domain shared by all receptor isoforms [58–60]. Homozygous (*fa*/*fa)* Zucker rats are obese and insulin resistant yet still respond to intracerebroventricular administration of leptin, albeit at higher doses compared to lean and obese rats in some reports [61–65]. Preserved effects of RYGB occurring in Zucker rats therefore demonstrate the ability of RYGB to overcome genetic leptin resistance, but not leptin deficiency, per se. Likewise, the well-recognized effects of RYGB in obese humans and rodents [4, 66] demonstrate its ability to overcome the acquired leptin resistance of diet- induced obesity. Our data therefore represent one of the first studies to describe the effects of RYGB in a model of complete leptin deficiency.

In summary, we demonstrate that leptin is dispensable for the weight-reducing and anorectic effects of RYGB. However, leptin is required for the effect of RYGB to improve glucose tolerance and insulin sensitivity. Our data also suggest that leptin may be involved in post-RYGB weight maintenance, possibly in the context of increased dietary energy content and intake with resultant weight regain as can occur in patients late after surgery. These data further define the underlying mechanisms of the beneficial effects of RYGB on body weight and glucose homeostasis and may facilitate the development of alternate, less-invasive yet similarly efficacious therapies than bariatric surgery.

## Abbreviations

DIO: diet-induced obese
HFD: high fat diet
MC4R: melanocortin-4 receptor
PF: sham, pair-fed sham
RC: regular chow
RYGB: Roux-en-Y gastric bypass
WM: sham, weight-matched sham
WT: wild-type
